# Dynamics of resilience of wheat to drought in Australia from 1991–2010

**DOI:** 10.1038/s41598-017-09669-1

**Published:** 2017-08-25

**Authors:** Jianjun Huai

**Affiliations:** 1 College of Economics and Management, Northwest A&F University, Yangling, Shaanxi 712100 China; 2 Western Development Research Center, Northwest A&F University, Yangling, Shaanxi 712100 China; 3 Laboratory of Ecosystem Prediction and Global Change, Northwest A&F University, Yangling, Shaanxi 712100 China

## Abstract

Although enhancing resilience is a well-recognized adaptation to climate change, little research has been undertaken on the dynamics of resilience. This occurs because complex relationships exist between adaptive capacity and resilience, and some issues also create challenges related to the construction, operation, and application of resilience. This study identified the dynamics of temporal, spatial changes of resilience found in a sample of wheat–drought resilience in Australia’s wheat–sheep production zone during 1991–2010. I estimated resilience using principal component analysis, mapped resilience and its components, distinguished resilient and sensitive regions, and provided recommendations related to improving resilience. I frame that resilience is composed of social resilience including on- and off-site adaptive capacity as well as biophysical resilience including resistance and absorption. I found that resilience and its components have different temporal trends, spatial shifts and growth ratios in each region during different years, which results from complicated interactions, such as complementation and substitution among its components. In wheat-sheep zones, I recommend that identifying regional bottlenecks, science-policy engagement, and managing resilience components are the priorities for improving resilience.

## Introduction

Ever since the theory of resilience was first proposed^1^, many studies have focused on resilience of the socioeconomic ecological systems (SES) related to climate change^2^. Resilience Alliance^3^ defined resilience as the ability of a SES to absorb shocks and regenerate after disturbances before exceeding the system’s threshold for stress tolerance. Resilience comprises resistance, absorption, and capacity for restoration that is sometimes called adaptive capacity (AC)^4^. Resistance, the capacity to withstand disturbance and recover from it, are both components of ecological resilience^5^. Capital theory links resilience to economics, sociology, and ecology^6^, and improving resilience is closely related to building human, social, and political capital^7^. In this sense, resilience theory provides a method to manage dynamics of the SES, which includes the adaptive management techniques related to sustainable development^8^. Consequently, enhancing resilience has been adopted as a systemic approach of embedding and integrating the multifaceted and articulated nature of vulnerability and resilience to climate disasters^9, 10^.

However, the complex relationship between resilience and AC has continued to be debated during the last decade. AC is the ability to reduce potential damage and take advantage of new opportunities^11^, and increasing AC is a critical part of improving the resilience of SES^12^. Any dynamic SES has a combination of resilience, adaptation, and vulnerability^13^, and AC affects the cycles of resilience and vulnerability simultaneously^14^. However, different adaptive options may impede the development of resilience^15^. Local adaptability may unintentionally reduce regional resilience and increase regional vulnerability^16^. An increase in AC of a SES in one region may cause another region to lose resilience^17^. The synergistic effects of the resilience and uncertainty of AC can make ecosystems more vulnerable to changes; therefore, the combination of active adaptive management and governance of resilience will require managers to examine the dynamics of AC and resilience^18^. The reasons for the complexity of the relationship between AC and resilience involve complementation and substitution among their various components in the context of the dynamics of resilience. The complementation comes from a positive causal relationship between indices and substitution from a negative causal relationship between indices^19^. For example, substitution among sources of the effects of output and competition for input affects the regional distributions of the impacts of climate change on dairy productivity in Australia^20^.

The dynamics of resilience refers to changes in resilience after shifts in temporal or spatial objectives, so it is difficult to define, measure, operate, and implement when the context changes. Additionally, resilience involves addressing issues at the conceptual, operational, and application levels. The conceptualization and applicability of resilience are still vague when used in the theory and practice of disaster risk management^21^; the simple definition of “resilience of what to what” conflicts with problems related to vagueness and malleability when compared to other measurements of resilience^22^. At an operational level, although each aspect of resilience has been expressed as a set of empirical indicators, measures of the totality of resilience is still unclear^23^, quantifying resilience is notoriously difficult that impedes to built through developing AC^24^. At the application level, the complicated relationship between vulnerability, resilience, and AC causes a gap between science and policy^25^. As a central concept to the accurate definition of resilience, the idea of “bouncing back” or interaction can be interpreted differently under three different narratives that can inspire different policies^26^. Therefore, dynamics of resilience cannot be clearly defined, measured, or implemented at spatial and temporal scales.

This paper aims to estimate resilience and assess the dynamics of resilience. I subsequently determine how to build resilience in different regions and at different times as constrained by local capital features. Specifically, the study has three main objectives. The first is to frame resilience, for which we used the case of resilience of Australian wheat to drought. The second objective is to examine the dynamics of resilience, that is, interactions such as substitution and complementation among resilience and its components at different times and in different regions. The third is to investigate the implications of such dynamics of resilience such as they may render different regions either resilient or sensitive and to assess the benefits of building resilience in a given region, so that I find an optimal resolution to developing regional resilience according to some regional capital constraints.

My work contributes to resilience research at the conceptual, operational, and application levels. First, I offer new synthesized method of studying resilience, combining sustainable livelihood framework (SLF) with the Principal Component Analysis (PCA) method through the use of a spatial map and a radar map. Second, this study shows that resilience is composed of social resilience including on- and off-site AC as well as biophysical resilience including resistance and absorption. Third, I found dynamic resilience to have different temporal trends, spatial shifts, and growth ratios in wheat-sheep zones from 1991–2010, which results from complementation and substitution between its components in the process of climate change. Overall, it provides useful information for local policy makers as well as for individual households designed to help them improve the resilience of wheat crops to drought.

## Research design

### Study areas

My study areas are all located within Australia’s wheat–sheep production zone (Fig. 1 in ref. 27). I analyzed the dynamic effects of resilience of wheat to climate change for several reasons.

First, Australia is believed to be the largest country among those most severely affected by climate change in the southern hemisphere. For instance, Australia is one of the world’s largest wheat exporters, and it has experienced long-term droughts since 1990^28^. Australia’s wheat–sheep production zone produces about 90% of the country’s wheat, but typically experiences a Mediterranean climate that can reduce wheat yield critically through affecting the wheat production areas^29^. For example, droughts in 2006–2007 caused wheat production to fall by roughly 61%^30^; wheat yield will be reduced by 5–25% in 2030 and 20% in the period of 2050–2100 because of the shorter duration of the growth period, which is expected to result from the changing climate^31, 32^. However, a 2014 Climate Change Performance Index published by the European Group Climate Action Network ranked Australia near to the bottom among all 34 countries in the Organization for Economic Co-operation and Development. This suggests that Australia needs improve the resilience of its wheat crop to climate change, actively and immediately.

Second, many studies have assessed the vulnerability of crops to drought in Australia’s wheat–sheep production zone^27, 33^, but few have calculated the resilience to climate change and estimate its dynamics. Australian wheat, as Australia’s most widely planted crop, is an appropriate product that can be used to assess the resilience of other crops to climate change, so we used wheat-sheep zones to assess the role of capital in improving AC^27^ and the role of capital in reducing vulnerability^34^. Based on both, I argue that the dynamics of the resilience of wheat to drought, which differs across regions and over time, is an interesting subject and requires urgent study. Meanwhile, Australian Agricultural and Grazing Industries Survey (AAGIS), provide data related to the resilience of Australian agriculture, which helped me to analyze the rural capital that supports rural livelihoods and the resilience of wheat to climate change in the wheat–sheep production zone (http://www.agriculture.gov.au/abares/surveys).

## Methodology

**Figure 1:**
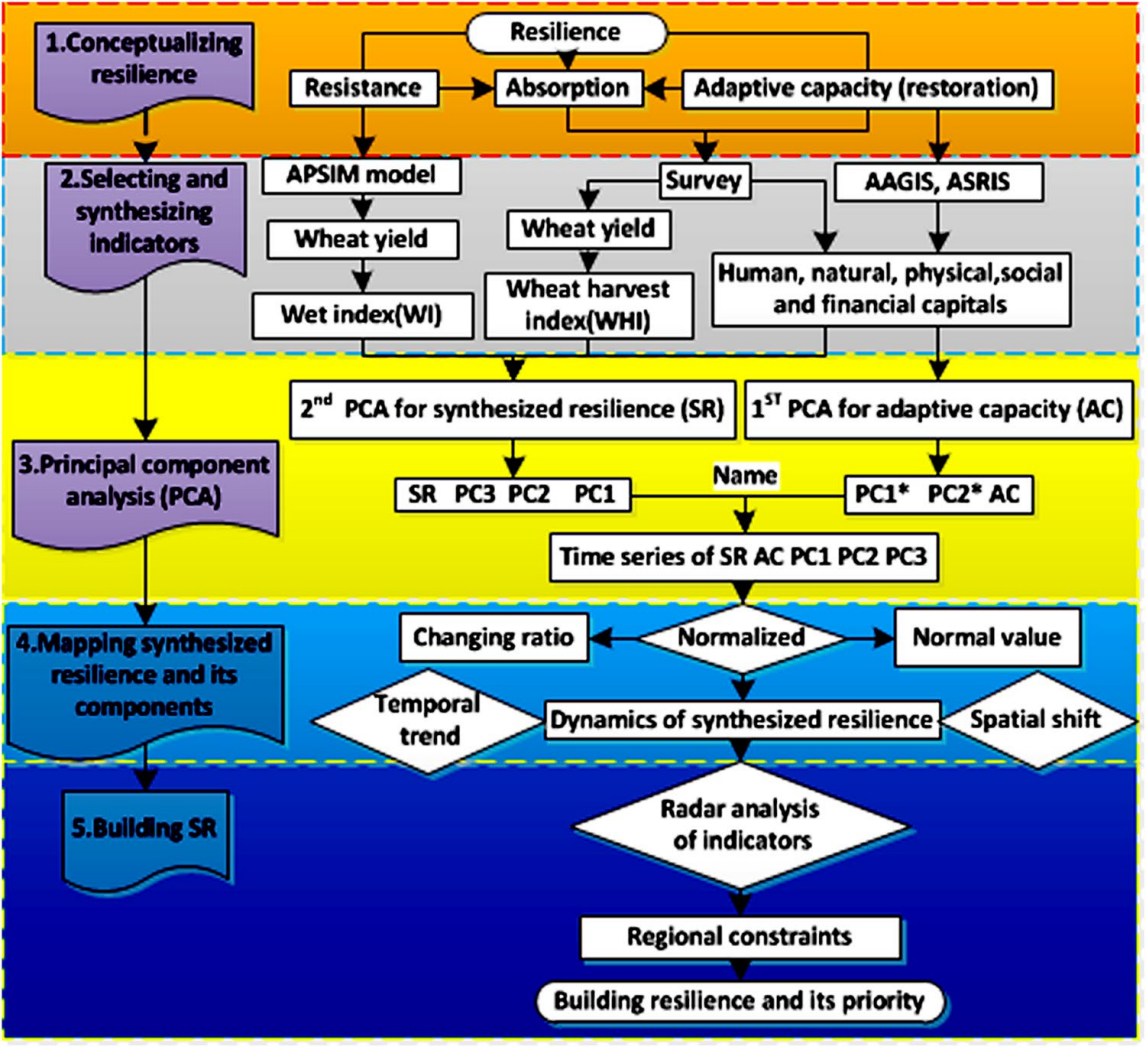
Five steps of analysis the dynamics of resilience.

The dynamics of resilience were assessed adopting the methodological work-flow illustrated in Fig. 1.

### Conceptualizing resilience

To capture the totality of resilience, I here framed resilience as a combination of social resilience including the on- and off-site AC and biophysical resilience that is composed of absorption and resistance. I used capital indicators from socioeconomic and ecological indicators from ecology. Biophysical resilience represents the magnitude of disturbance a system can handle before moving into a different state^35^. Social resilience is defined as the ability of human communities to withstand external shocks^23^. In wheat-drought resilience, resistance refers to the ability of wheat to tolerate drought, as represented by the benefits from the humid climate; absorption denotes crop mitigation to drought as measured from actual wheat yield; restoration refers to the ability of the crop to recover its pre-stress status, as determined by farmer’s AC, which incorporates human, social, natural, physical, and financial capital.

### Selecting indicators: Measuring resistance, absorption, and restoration

Most measurements of resistance and absorption of crop to drought have been found to be related to the crop yield and drought in previous works. For instance, the stress susceptibility percentage index (SSPI), relative drought index (RDI), abiotic tolerance index (ATI), stress non-stress production index (SNPI), and drought resistance index (DI) are considered the most suitable indicators for screening drought-tolerant cultivars^36^. In arid and semi-arid regions, beneficial drought resistance indicators, such as canopy temperature, stomatal resistance, and rate of water loss, have a significant relationship to the yield reduction ratio^37^. In northern China, drought index is the most comprehensive drought resistance index, and it also includes the opposite yield (drought resistance coefficient) and absolute yield of the wheat variety^38^. Therefore, I predicted wheat yield under special climatic conditions to assess climate change, and actual yield in the past few decades to determine the reductions in yield due to the drought. I measured wheat–drought resistance using a wet index (WI) calculated using predicted wheat yield and wheat-drought absorption using wheat harvest index (WHI) measured by actual wheat yield. The predicted wheat yield is weighed by the area sown to wheat (produced) in the National Land Use Map of Australia in 2006, and were simulated at 50 kg N ha^−1^ from 1991–2010 using APSIM version 7.3^39^.

Under the SLF, restoration or AC is conceptualized as the diverse forms of livelihood capital, such as human, social, natural, physical and financial forms of capital^25, 27, 40, 41^. As the stock of knowledge, talent, skills, and abilities of farmers, human capital includes labor productivity expressed by total labor used (TLU) on a farm, and management ability expressed by the age of a manager (AM). Social capital is an appreciable and convertible long-lived asset and collective good that has relationships with other actors that need maintained continuously. Its effects flow from the information, influence, and solidarity it makes available to the actor^42^. Social capital is mainly embedded in the social networks between individuals and organizations; annual telephone charges (TC) are an indicator of social connections and knowledge sharing. Natural capital refers to some natural resources that supply environmental services and goods, such as water and erosion control; here, its proxies are crop productivity (crop-revenue level: CRL) and the soil water-holding capacity (SWHC). Physical capital, the long-term existence form of production materials, here includes land value and improvements (LVI) and expenditure for electricity (EE). Like any kind of money, financial capital combines total closing capital (TCC), access to financing (AF) and total cash income levels (TCIL) (Table 1).

**Table 1:** Definitions and measurements of resilience.

Composite	Indicator	Definitions or Measurements (Unit)
Resistance	Wet index (WI)	Ratio of predicted wheat yield (PWY) in region r and year y to its mean. A WI that is greater or less than one expresses a predicted return or loss in the wet or dry years.
Absorption	Wheat harvest index (WHI)	Ratio of actual wheat yield (WY) in region r and year y to its regional average. WHI had a score greater or less than one for years with good or poor harvests.
Restoration (Adaptive Capacity)	Total labor used (TLU)	Total number of full-time weeks worked by all farm workers including hired labor (week).
	Age of manager (AM)	Age of the primary decision-maker in the farm business (year).
	Telephone charges (TC)	Telephone charges per year ($K).
	Value of land and improvements (VLI)	Market value of all land operated and fixed improvements starting at the end of the financial year estimated by the owner–manager or cooperator in the survey year ($M).
	Electricity expenditure (EE)	Expenditure on electricity per year ($K).
	Crop-revenue levels (CRL)	Crop-revenue levels in the n^th^ year equal to the average of summing total crop gross revenues in the previous n − 1 years. The total gross revenues come from sales of crops and hay ($K).
	Soil water-holding capacity (SWHC)	Drained upper limit minus crop lower limit. Drained upper limit is the amount of water that a particular zone of soil holds after drainage has largely ceased. Crop lower limit is the amount of water remaining after a particular crop has extracted all the water available to it from the soil zone (mm).
	Total closing capital (TCC)	Closing value of all assets used on the farm including leased equipment but excluding machinery and equipment either hired or used by contractors ($M).
	Access to financing (AF)	Borrowing capacity plus liquid assets. Borrowing capacity is derived from each farm’s equity ratio. When the equity ratio is less than 70%, borrowing capacity is zero; otherwise borrowing capacity = (equity ratio − 0.70) × total closing capital ($M).
	Total cash income level (TCIL)	Total cash income level in the n^th^ year is the averages of summing total cash income in the previous n − 1 years. Total cash income equals that cash income plus off–farm income ($K).

1$K, 1000$; 1$M, 1000,000$; Data Source: Predicted wheat yield comes from the Commonwealth Scientific and Industrial Research Organization (CSIRO); Soil water-holding capacity is from Australian Soil Resource Information System (ASRIS); others are from Australian Agricultural and Grazing Industries Survey (AAGIS). The survey year is 1991–2010.

### Synthesizing resilience by PCA

PCA is an effective regression method used to reduce the number of variables in the assessments of vulnerability to climate change, so, here, resilience and AC were synthesized by two PCAs. I tested the suitability of all indicators in Table 1 for PCA and determined the sufficient number of factors through parallel analysis. Naming each principal component (PC), I then computed AC and resilience according to their own factor scores and responding initial Eigenvalues, as shown in Equations (1)–(2).

1 $$P{C}_{r,y,i}=\sum _{r,y}^{m,k}\,{x}_{r,y}\times (\frac{{\alpha }_{r,y}}{\sqrt{{\delta }_{i}}})$$

2 $${Y}_{r,y}=\sum _{i=1}^{n}\,P{C}_{r,y,i}^{Y}\times CP{V}_{r,y,i}^{Y}$$

where r represents the survey region, y represents the survey year, i represents the number of the PC, δ is the initial Eigenvalues for each PC, x is each indicator in Table 1, and a is the constituent variables for each component. CPV signifies the contributive percentage of explained variances, Y represents AC or synthesized resilience (SR).

### Mapping synthesized resilience and its components

Rather than reporting the indices statically and directly^43^, spatial mapping based on PCA can reflect spatial distribution by aggregating multiple social–ecological indicators into intuitive resilience indices^44^. Additionally, comparisons of different spatial maps in a time series can be used to illustrate the dynamics of the resilience. After normalizing each PC, AC, and SR on a scale of 0–1 in Equation (3) and using the ArcGIS (Version 11.2) software package, I created geographical maps for every five-year period:

3 $$\varepsilon =\frac{A-{A}_{\min }}{A-{A}_{\max }}$$

where ε is the normalized value, A is the average of each PC, AC, SR, A_min_ and A_max_ are the minimum and maximum values of A.

**Figure 2:**
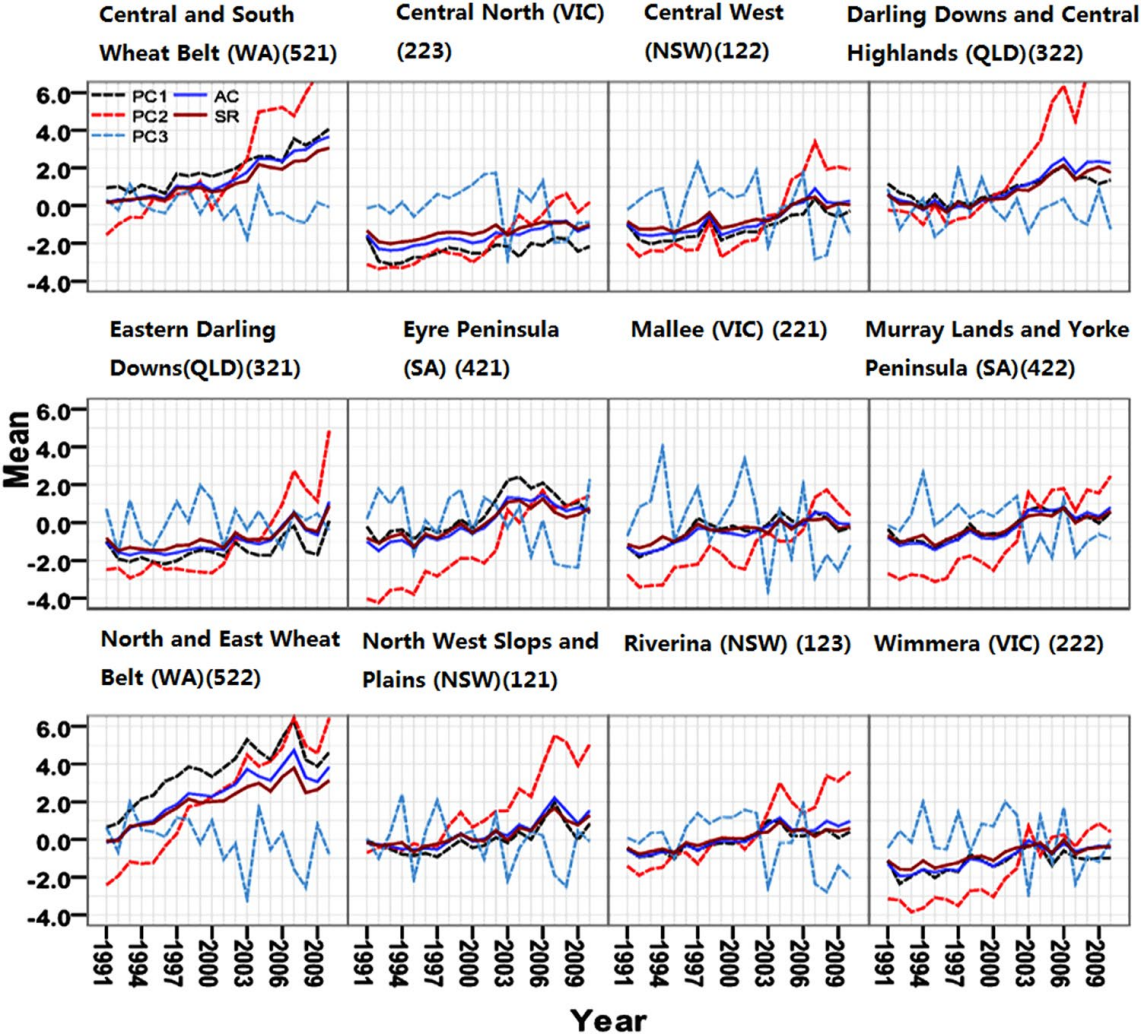
Time series of annual resilience (SR) and its components in 1991–2010 in Australia’s wheat–sheep production zone. The numbers in parentheses represent the regional code. For instance, 121 is the regional code for the North West Slopes and Plains.

**Figure 3:**
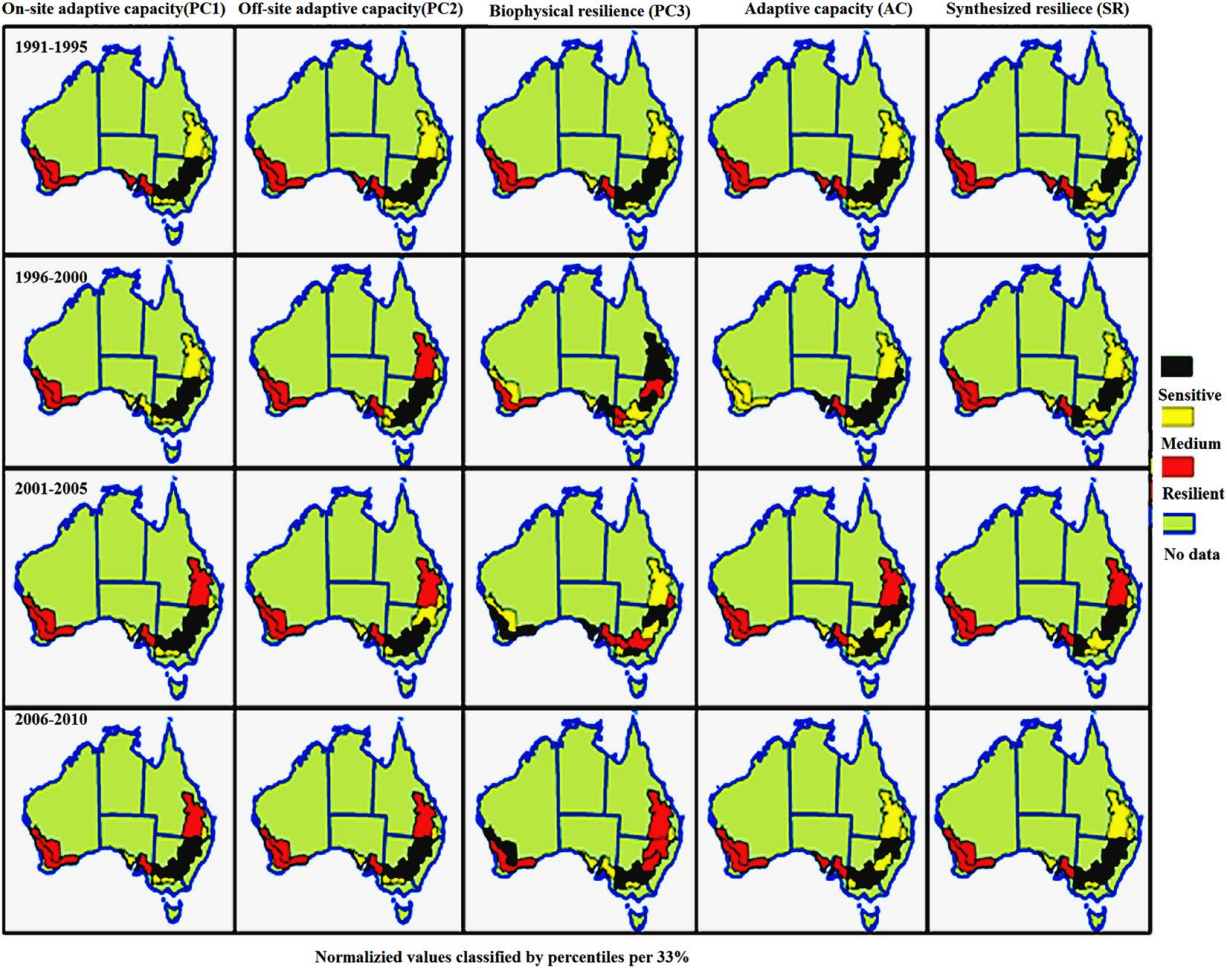
Regional resilience and components every five years in the wheat–sheep production zone in Australia. Resilient cases are the regions with top 33% of metrics (red), sensitive cases are the regions with the bottom 33% of metrics (dark red), and the other 33% are neutral (yellow). The map used and modified here is based on the one in Fig. 1 in our previous study (doi:10.1371/journal.pone.0117600)^27^, which is an open access article distributed under the terms of the Creative Commons Attribution License, and which also permits unrestricted use, distribution, and reproduction in any medium, provided the original author and source are credited. Jianjun used ArcGIS (version 11.2, http://www.esri.com) to create Fig. 3.

**Figure 4:**
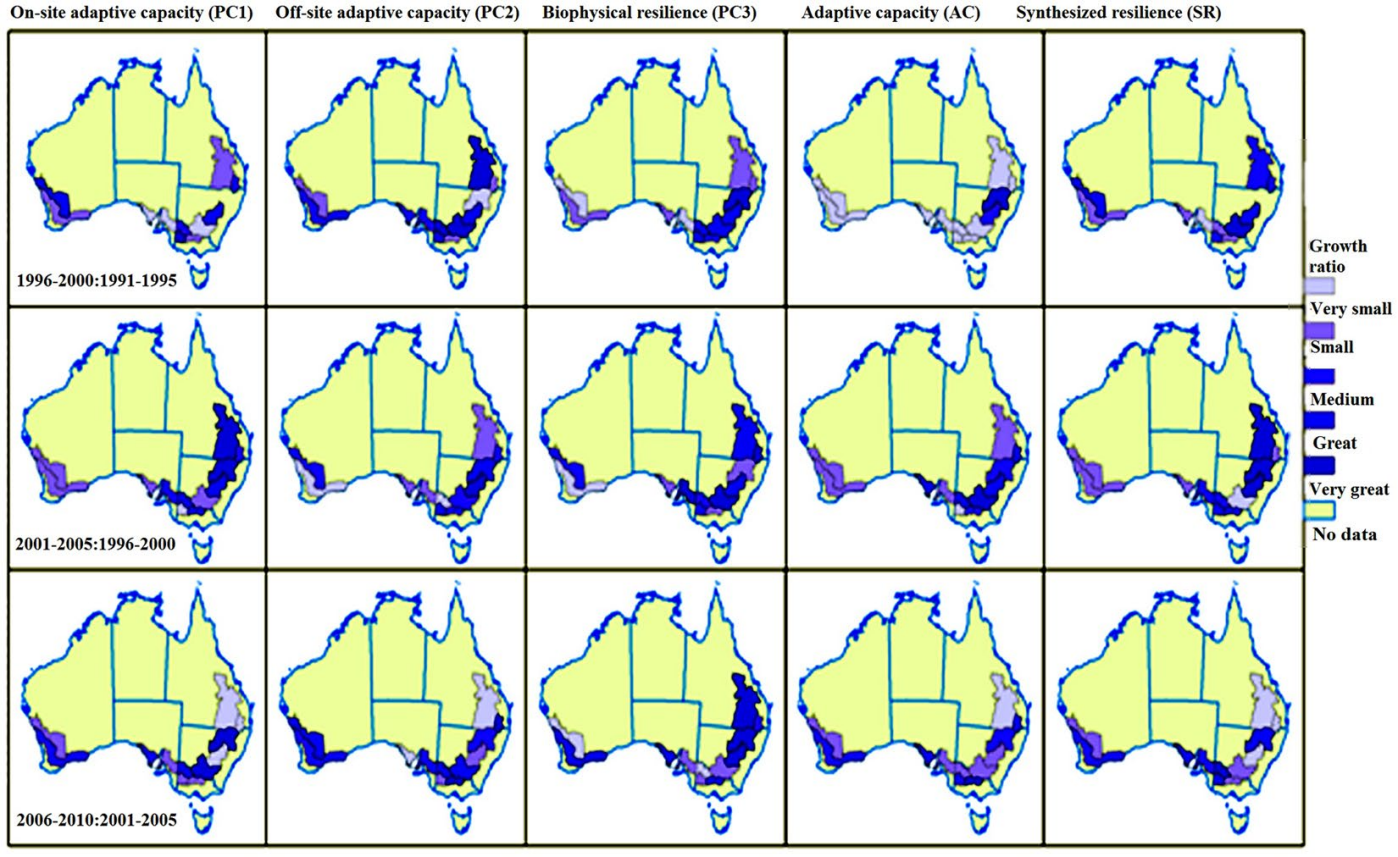
Growth ratios of resilience and its components. “1996–2000:1991–1995” means the ratio of average of each metric (here is PC1,PC2, PC3, AC and SR, respectively) in 1996–2000 to its average in 1991–1995; For instance, the map of SR in “1996–2000:1991–1995” represents the ratio of average SR during 1996–2000 to the average SR during 1991–1995. Similar ratios include “2001–2005:1996–2000” and “2006–2010:2001–2005”. The map used and modified here is based on the one in Fig. 1 in our previous study (doi:10.1371/journal.pone.0117600)^27^, which is an open access article distributed under the terms of the Creative Commons Attribution License, and which also permits unrestricted use, distribution, and reproduction in any medium, provided the original author and source are credited. Jianjun used ArcGIS (version 11.2, http://www.esri.com) to create Fig. 4.

To understand the dynamics of resilience, I used Fig. 2 to show time series of annual resilience (SR) and its components in 1991–2010 in Australia’s wheat–sheep production zone. The percentile method was used to define the resilient or sensitive regions in each map in Fig. 3. In Fig. 4, I firstly calculated the growth ratio of average of each metric that equals to the ratio of each average SR, AC and their components in five years to its average in last five years, respectively; For instance, the map of SR in “1996–2000:1991–1995” represents the ratio of average SR during 1996–2000 to the average SR during 1991–1995. I then classified 12 regions into five types through mapping five classes of the growth ratios of each PC, AC and SR, where the smaller growth ratio indicates slower development. Therefore, I here define the first one-fifth of regions as the slow developing region, then the second fifth is the slow-middle-developing regions, the third fifth the middle-developing region, and so on, through the top fifth, which represents the quickest-developing region. The shifts in temporal and spatial trends of these five classes reflect the dynamics of resilience from 1991 to 2010.

### Building synthesized resilience

The interactions between different PCs represent the dynamics of resilience; the trade-offs between complement and substitution of each capital commonly influence the function of adaptive capacity. So I analyzed the interactions between each PC and indicators to find the optimal methods for improving resilience. I then used a radar map to depict the constituent indicators every five years allowing us to show the regional constraints on improving resilience and the priorities of adaptive options for each region.

## Results

My results showed the composites (Table 2), time series (Fig. 2), absolute change (Fig. 3) and growth ratios (Fig. 4) of each PC, AC, and SR from 1991–2010 in Australia’s wheat–sheep production zone.

**Table 2:** Principal component analyses (PCA) of adaptive capacity and resilience.

Items	Adaptive capacity^a^		Resilience^b^		
Variables	PC1^AC^	PC2^AC^	PC1^SR^	PC2^SR^	PC3^SR^
CRL	0.871	0.150	0.873	0.144	
TC	0.823	0.456	0.823	0.455	
TLU	0.821	0.151	0.820	0.152	
TCIL	0.810	0.396	0.813	0.389	
SWHC	−0.659		−0.660		
VLI	0.246	0.938	0.250	0.935	
AF	0.254	0.934	0.258	0.931	
TCC	0.298	0.928	0.302	0.925	
AM	−0.439	0.656	−0.435	0.650	
EE	0.382	0.474	0.381	0.477	
WI					0.865
WHI					0.858
% of Variance	52.91	21.37	44.25	18.31	11.85
Initial Eigenvalues from PCA	5.29	2.14	5.31	2.20	1.42
Random Eigenvalue from parallel analysis	1.36	1.24	1.40	1.30	1.21
Kaiser–Meyer–Olkin Measure of Sampling Adequacy.	0.74		0.74		
Bartlett’s Test of Sphericity	Approx. Chi-Square	3576.03			3635.19
	Df. (Sig.)	45(0.000)			66(0.00)

All short forms are shown in Table 1; Extraction Method, Principal Component Analysis; Rotation Method, Varimax with Kaiser Normalization; ^a^Rotation converged in 3 iterations; ^b^Rotation converged in 4 iterations.

### Synthesis of adaptive capacity and resilience

In Table 2, the Kaiser–Meyer–Olkin value is greater than 0.7, Bartlett’s Tests of Sphericity is significant at 1%. These findings show that the variables are available for PCA. The retained PCs explain roughly 74% of the variation of AC and SR, which means that their components can explain 74% of the changes in AC and SR. For either AC or SR, the first PC (PC1) represents the characteristics of cropping, communication, labor-investing, financial security and liquidity, land conditions in the on-farm society; the second PC (PC2) reflects the features of technological improvements, credit exchange, financial managements, human capital and infrastructure developments in the off-farm society; the third PC (PC3) that is only for SR reflecting resistance and absorption of wheat to drought. In all, adaptive capacity consists of PC1^AC^ and PC2^AC^, while synthesized resilience consists of PC1^SR^, PC2^SR^, and PC3^SR^, here called social resilience (including on- and off-site adaptive capacity) and biophysical resilience, respectively.

### Dynamics of resilience

At the operational level, the dynamics of resilience can be expressed by temporal and spatial shifts of resilience and its components in the maps. Figure 2 illustrates from 1991 to 2010 in wheat-sheep zones the rising AC overlapped with the SR of each region throughout the surveyed years, and PC3 exhibited greater fluctuations in each region than PC1 and PC2, which shows that the risk of a deteriorating natural environment from PC3 is higher than that for social environment.

The trends of SR, AC, PC1, PC2, and PC3 have complicated change every five years in the longitudinal view of the Fig. 3. For instance, PC3 rises in the northern and eastern wheat belts but has a fall-rise-fall pattern in northwestern slopes and plains; AC increases in West Australia and Central West but decreases in Darling Downs and Central Highland. The spatial distributions of SR, AC and their components at five-year intervals (Fig. 3) are similar to the annual trends of time series of resilience (SR) and its components in 1991–2010 in each region. The higher AC and SR, PC1, and PC2 appeared in the wheat–sheep production zones of WA that is located in resilient regions; while the smaller of them mainly occurred in NSW and Victoria (VIC), which are sensitive regions; during the past twenty years the resilient regions for PC3 have shifted from southwest to northeast, such as from WA to the Central and Southern Wheat Belt, then to QLD.

Each PC, SR, and AC increased absolutely from 1991 to 2010, with different growth ratios in different regions at different stages (Fig. 4). The higher growth ratio of AC occurs in the Northwest Slopes and Plains (1996–2000), Riverina (2001–2005), and North Central regions (NSW) (2006–2010). Meanwhile, PC1, PC2 and PC3 increase at varying ratios every five years in different regions. Comparing the spatial and temporal trends of each index, I established resilient, neutral, and sensitive classifications, three unequal distributions (Fig. 3) and observed many levels of speeds of development for each component in each region for different five-year stages (Fig. 4). The biophysical resilient regions shifted from southwest to northeast, while the sensitive regions moved from northeast to southwest, so the same spatial–temporal trends of AC have been observed in the sensitive regions (Figs 3 and 4). My classifications of resilient and sensitive regions strongly support the claim that agricultural operations in the Western, Northern, and Eastern Wheat Belts in Australia are resilient to climate change^45^.

## Discussion

In the study, a new synthesized method of studying resilience was used to combine SLF with the PCA method and geographical information systems (GIS) and ARCmap. Specifically, I (1) provide a framework for assessing resilience that includes AC, resistance, and absorption, (2) use principal component analysis (PCA) to integrate resistance, absorption, and restoration (AC) into resilience, (3) map the spatial and temporal dynamical trends of resilience and its components, (4) classify the resilient and sensitive cases according to annual and five year indicators in each region, and (5) recommend building components to improve resilience based on regional constraints. The PCAs integrate each indicator into principal components (PCs) that are then decomposed into executable policies. The geographical maps of resilience, AC, and their PCs can provide useful information that will allow householders to understand the dynamics of resilience and the interactions between their components. More detailed radar maps of livelihood capital can improve the targeting and effectiveness of local policies of the government and livelihood strategies of households by showing the local constraints in each region. Comparisons of different spatial maps of PCs and radar mapping of the livelihood capital can help determine the rules controlling resilience as it relates to climate change. There are at least five advantages of this synthesized methodology. It (1) illustrates the rules governing spatial and temporal shifts in sharply visual ways, (2) matches the integration and typology of resilience and its components, (3) explains substitution and complements between different indexes by comparing lines and maps, and (4) provides useful information for decision-making at both the household and policy levels.

This research highlights the fact that resilience is driven by the complement and substitution among all of its components. These interactions occur in the process of climate change and affect the wheat productions over time at different scales. The trade-off of complement and substitutions of PC1, PC2, and PC3 determine resilience at different periods and regions. For example, in the Darling Downs and Central Highlands (QLD), the substitution between high PC1 and medium PC2 initiates the neutral AC during 1996–2005, while the complement between high PC1 and high PC2 aggregates into high AC during 2006–2010 (Fig. 3). The substitutions among different regions confirmed that increasing adaptability unintentionally reduces resilience^17^; for instance, rising ratios of PC3 in the Central and Southern Wheat Belt caused a decrease ratios of PC3 in the Northern and Eastern Wheat Belts of West Australia in 1996–2005 (Fig. 4). The substitutions between PCs also low growth ratios of SR. For example, the relatively small growth ratio of PC1 trades off for the much great growth ratio of PC2 in Downs and the Central Highlands (QLD) during 1996–2000, which results in the small growth ratio of SR (Fig. 4). The complementary effects create higher growth ratios of some components at the same periods and regions; for instance, in Fig. 4 the small ratios of PC3 complement smaller ratios of AC into a relative higher growth ratio of SR in Downs and the Central Highlands (QLD), during 1996–2000.

At the application level, improving resilience is required to interrelate the biosphere with a society and to create flexible and innovative collaborations, through management diversification, building human capacity, and reinforcing social networks^46^. In Australia, identifying regional bottlenecks, science-policy engagement, and managing resilience components are the priorities for improving resilience.

**Figure 5:**
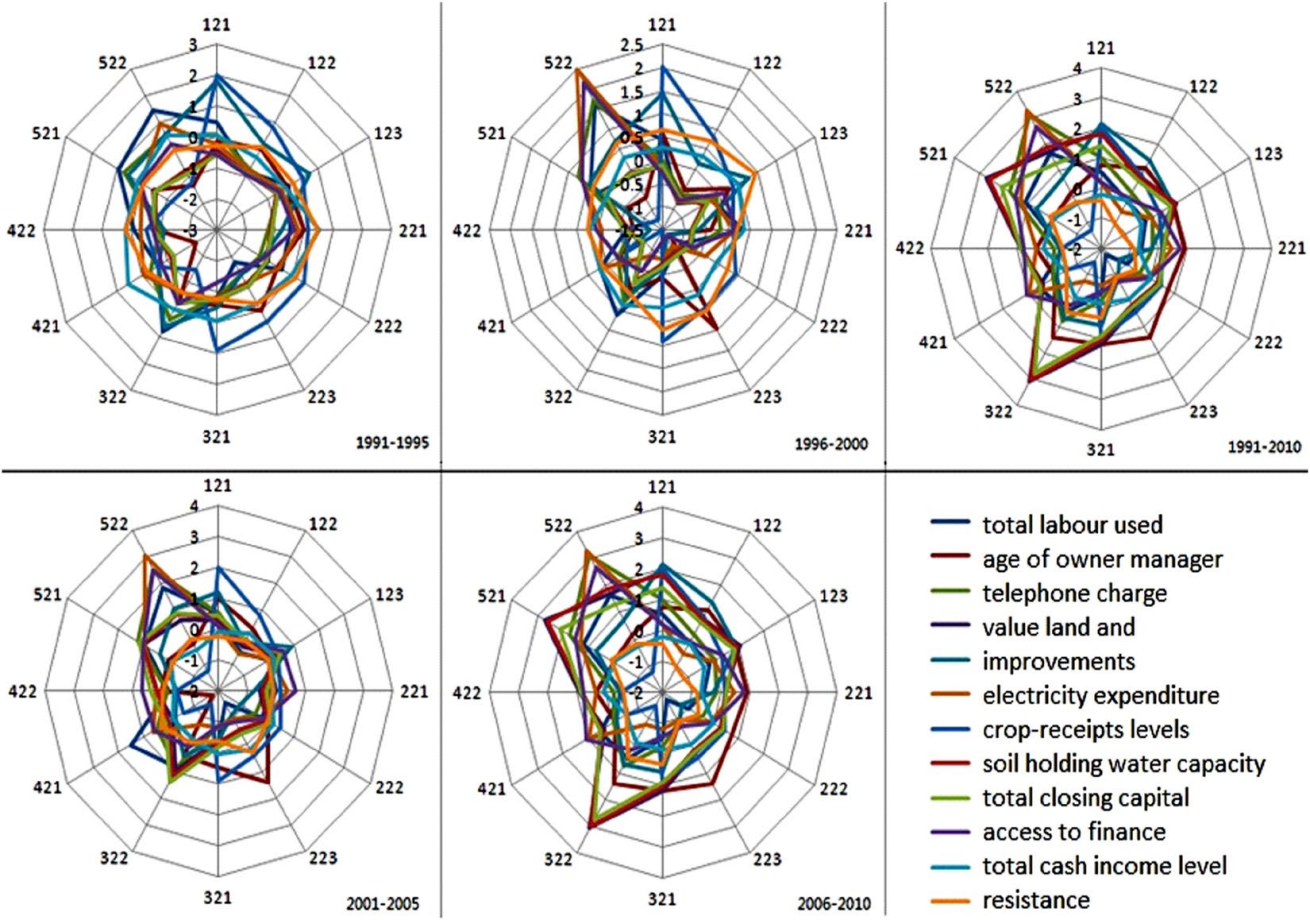
Radar maps of average components every five-year that show the regional bottlenecks of improving resilience. The number by each branch of Radar is region code that is gained from Fig. 1 in a previous work^27^. The smaller indicators in each region represent the regional constraints every five year.

When strengthening the components of resilience, decision makers first need to identify any regional bottlenecks. From a macro perspective, pressure on declining trade and increasing urbanization^25^, and other regional bottlenecks, to some extent, block the development of resilience in the wheat–sheep production zone. From a micro perspective, each region has different dynamics of resilience and its components. I used a radar map to depict the constituent indicators every five years, allowing me to show the regional constraints on improving resilience and the priorities of adaptive options for each region. If resilience is constrained by specific aspects of livelihood capital for households, the government should develop stronger and more effective policies and programs to reduce the adverse effects of these local constraints. The Radar map further shows each region had different average compositions from 1991 to 2010 (Fig. 5). I found that different bottlenecks limit the development of indicators in different times at different regions. For example, weak resistance is limited in the Central North region of Victoria, while low soil water-holding capacity constrained resilience in the Darling Downs and Central Highlands in 1991–2010. The common bottlenecks in the wheat–sheep production zone include average income, soil water-holding capacity, electricity consumption, total labor used on farm, and age of the land manager (Fig. 5). I recommend each region strike a balance among these components to resolve their current bottleneck issues. Identifying and overcoming regional bottlenecks and allowing land managers to balance local development provide a good way to prioritize improving resilience and AC. For example, the Central North region of Victoria needs greater resistance and Darling Downs and Central Highlands need better soil water-holding capacity.

Creating scientifically sound policies to manage the components of resilience (e.g., resistance, absorption, and AC) will help local stakeholders deal with climatic disasters. Biophysical resilience benefits from improving resistance and absorption in the environment^47^. The interactions between different PCs represent the dynamics of resilience; the trade-offs between complement and substitution of each capital commonly influence the function of AC. Findings from the substitution and complementation between each composite can be useful inputs for developing a combination of policies that are designed to strengthen resilience. Some mitigation policies (e.g., cultivating drought resistant varieties) can augment the resistance of wheat to drought. Selecting efficient technologies (e.g., conserving crop-diversity) can improve the tolerance of wheat to drought. Managing AC depends on developing human, physical, social, natural, and financial capital in socioeconomic contexts. For example, specific investments in education and health services can increase human capital, and programs that support community development and communication infrastructure, such as Australia’s land care movement, can enhance social capital. Policies that accelerate rural development and expand access to the markets can create better physical infrastructure and provide additional market opportunities^48^.

The science-policy gap also should be resolved by managing the components of resilience. To make science more relevant to policy, resilience assessments need to inform action by and on behalf of stakeholders to increase resilience and build AC. The government, scientists, and non-government organizations should provide farmers with a decision-support system, seasonal weather, and agricultural forecasting as well as information related to the effects of climate change on farming systems^49^.

## Conclusions

Although enhancing resilience is a well-recognized adaptation to climate change, little research has been undertaken on the dynamics of resilience. This occurs because complex relationships exist between AC and resilience, and some issues also create challenges related to the construction, operation, and application of resilience. This study identified the dynamics of temporal, spatial changes of resilience found in a sample of wheat–drought resilience in Australia’s wheat–sheep production zone during 1991–2010.

Combining SLF with the PCA method through spatial resilience mapping, I provide a synthesized methodology of resilience, expand knowledge on the dynamics of resilience, and recommend effective strategies of improving resilience, which are useful and urgently needed for resilience conceptualization, operation, and application. I conclude that a synthesis of resilience is needed to create an interaction between social and biophysical resilience due to the substitutions and complementation events among their components, recommend that preferentially solving regional constraints, building components of resilience, and trading off the related components are the important steps required to improve resilience.

However, the present analysis has some limitations. First, the measurements of the resilience of wheat yield to drought lost more information on the decision-making process about improving resilience. I will measure resilience of agricultural profit to climate changes. Second, it exemplifies here how complements and substitutes between each PC affect resilience; these complex interactions may reduce the effectiveness of the SLF in resilience assessments. The tradeoff among these interactions will be an interesting topic for future studies.
